# Glaucoma Syndromes: Insights into Glaucoma Genetics and Pathogenesis from Monogenic Syndromic Disorders

**DOI:** 10.3390/genes12091403

**Published:** 2021-09-11

**Authors:** Daniel A. Balikov, Adam Jacobson, Lev Prasov

**Affiliations:** 1 Department of Ophthalmology and Visual Sciences, University of Michigan, Ann Arbor, MI 48105, USA; dbalikov@med.umich.edu (D.A.B.); adajac@med.umich.edu (A.J.); 2 Department of Human Genetics, University of Michigan, Ann Arbor, MI 48109, USA

**Keywords:** juvenile open-angle glaucoma, pediatric glaucoma, Singleton–Merten syndrome, Aicardi–Goutieres syndrome, aniridia, mucopolysaccharidosis, Peter’s anomaly, osteogenesis imperfecta, Stickler syndrome, nanophthalmos, anterior segment dysgenesis, Axenfeld–Rieger syndrome

## Abstract

Monogenic syndromic disorders frequently feature ocular manifestations, one of which is glaucoma. In many cases, glaucoma in children may go undetected, especially in those that have other severe systemic conditions that affect other parts of the eye and the body. Similarly, glaucoma may be the first presenting sign of a systemic syndrome. Awareness of syndromes associated with glaucoma is thus critical both for medical geneticists and ophthalmologists. In this review, we highlight six categories of disorders that feature glaucoma and other ocular or systemic manifestations: anterior segment dysgenesis syndromes, aniridia, metabolic disorders, collagen/vascular disorders, immunogenetic disorders, and nanophthalmos. The genetics, ocular and systemic features, and current and future treatment strategies are discussed. Findings from rare diseases also uncover important genes and pathways that may be involved in more common forms of glaucoma, and potential novel therapeutic strategies to target these pathways.

## 1. Introduction

Glaucoma comprises a group of disorders characterized by optic neuropathy resulting from retinal ganglion cell death in a specific pattern. Glaucoma is generally classified into two main categories: open angle, in which the iridociliary drainage angle is open, and angle closure glaucoma, in which the angle is obstructed. Though there are many risk factors contributing to glaucoma, including age, sex, ethnicity, blood pressure, medications, and elevated intraocular pressure, there is an additional strong genetic component [1]. Because of its progressive and irreversible nature, glaucoma is the leading cause of permanent vision loss worldwide [2]. At present, nearly 60 million people worldwide are thought to have glaucoma. This number is likely an underestimate as glaucoma is underdiagnosed in certain populations [3,4].

Childhood glaucoma comprises a small, but significant fraction of the total population of glaucoma patients [5]. Glaucoma affecting infants and children, including primary congenital glaucoma (PCG) and juvenile open-angle glaucoma (JOAG), is estimated to affect 1:10,000 to 1:100,000 children worldwide [6,7,8], with higher incidence in areas with increased consanguineous marriages [9]. It represents a high disease burden, contributing to 5–18% of childhood blindness [5]. Childhood glaucoma may be more difficult to diagnose given that standard ophthalmic testing performed for glaucoma is difficult to perform in children, and the precise pathogenic mechanisms are difficult to define. Angle procedures such as goniotomy or trabeculotomy are typically the first line treatments, but these are often ineffective in some subtypes if downstream outflow pathways are affected, as is common in many secondary glaucomas. Many childhood glaucomas are associated with monogenic systemic syndromes, which can offer important clues into the etiology and mechanisms of this group of diseases by identifying specific genes and pathways important in ocular structure and function. In this review, we outline several broad classes of childhood glaucoma that have syndromic presentations, discuss their underlying genetic findings, and present the state of the art and limitations for diagnosis and treatment.

## 2. Syndromes

### 2.1. Anterior Segment Dysgenesis Syndromes

#### 2.1.1. Axenfeld–Rieger Syndrome

Axenfeld–Reiger syndrome (ARS) was first documented as a specific constellation of pathologies in 1920 by Axenfeld but later addended by Rieger in 1934 [10,11]. Two predominant genes, *paired-like homeodomain 2* (*PITX2)* and *forkhead box C1* (*FOXC1*), have been described to cause the vast majority of ARS (nearly 60% of all cases) [12,13,14,15]. *PITX2* and *FOXC1* both encode transcription factors that regulate gene expression during eye development [16,17,18] with more than 80 pathogenic variants (deletions, insertions, splice variants) in *PITX2* and 50 pathogenic variants in *FOXC1* have been described in ARS [19,20,21]. Both are inherited in an autosomal dominant manner with variable penetrance and the central pathogenic mechanism is thought to be haploinsufficiency for these transcriptional factors [22,23]. Curiously, a key study by Berry and colleagues discovered that both *PITX2* and *FOXC1* proteins interact with and regulate key developmental pathways (the former negatively impacting the latter) [24].

Classic ocular findings in ARS include bilateral posterior embryotoxon (the anterior displacement Schwalbe’s line, the junction of cornea and sclera), corectopia, iris thinning, polycoria, and abnormal angle structures (Table 1) [25]. Nearly 50% of children develop glaucoma [10,26], and several more recent studies have demonstrated that glaucoma occurs in 20–74% of patient subjects with *PITX2* pathogenic variants and 44–100% of patient subjects with *FOXC1* pathogenic variants [15,27,28]. Of note, iris hypoplasia was originally described by Berg in 1932 in a family with thin irides and a very high incidence of glaucoma (ages 16–43) [29]. Iris hypoplasia, over time, has been considered to be part of ARS due to the significant syndromic overlap with ARS and pathogenic variants in both *PITX2* [30] and *FOXC1* [12]. Systemically, ARS can have extraocular manifestations, the most commonly observed being facial and dental anomalies (e.g., dental hypoplasia, flat mid-face) [31], umbilical abnormalities [32], and pituitary abnormalities (e.g., empty sella syndrome and growth hormone deficiency) [33,34]. Less common, but known, syndromic associations include SHORT syndrome [35], short FRAME syndrome [36], cardiac defects [37], sensorineural hearing loss [38], and myotonic dystrophy [39]. Sensorineural hearing loss may be a more prevalent feature of *FOXC1* mediated ARS [40]. The significant genetic heterogeneity and phenotypic variability of this condition may be attributable to stochastic events during ocular development and systemic organogenesis, with many genes and precise gene timing and dosage needed for proper anterior segment development.

Patients suspected of having ARS are always recommended to undergo genetic testing. Many common methods include chromosome analysis and DNA array comparative genomic hybridization; a modern bioinformatics analysis significantly improved detection efficacy [41]. Pathogenic variants in ARS genes *FOXC1* and *PITX2* make up about 40% of ASD cases [42], but the diagnostic yield is likely higher in patients with classic ARS. Because of the complexity of the disease, there are currently no clinical trials underway for the treatment of ARS. However, clinical management does exist. Many first line therapies include glaucoma eye drops, but this is not always effective given the angle structure (as demonstrated in a retrospective study [27]). In most cases, surgical interventions to modify or bypass the angle, such as trabeculectomy or tube shunt, are the remaining options for these patients [43].

#### 2.1.2. Peters Anomaly

Peters anomaly (PA) is a heterogenous genetic disorder associated with at least eight different genes [44]. The most common genes include *PITX2* [14], *FOXC1* [12,13], *PAX6* [45,46], *CYP1B1* [47], and *B3GALTL* [48]. Inheritance of gene pathogenic variants for PA are also heterogenous with autosomal dominant, autosomal recessive, and sporadic patterns of inheritance [49,50]. It is believed that failure of the lens separation from the surface ectoderm during early eye development gives rise to the ocular manifestations of the disease [51]. Clinically, many ocular findings for PA can be subtle. With careful ophthalmic examination, patients can have unilateral or bilateral absence of corneal endothelium, absence of the Descemet membrane, central corneal opacities, and iris synechiae [50,52]. Patients with PA have an incidence rate of glaucoma of nearly 50%, although it is rarely found at birth. With respect to syndromic manifestations, extraocular findings are defined in Peters Plus, which includes short stature, abnormal ears, brachydactyly [53], cleft lip/palate, facial dysmorphia, Potter syndrome, dextrocardia, and hydrocephalus [54,55,56,57]. 

Treatment for PA is also limited to the same treatments as ARS. As reviewed by Dolezal and colleagues, many patients require surgical intervention in order to address intraocular pressure [58]. Surgeries employed include trabeculotomy, trabeculectomy, tube shunts, as well as cycloablation of the ciliary body. However, the success rates for surgical intervention rarely exceed 50% (0% trabeculotomy, 25% trabeculectomy, and 53% for tube shunt), suggesting that tube shunts are the optimal conventional treatment, but novel approaches are being attempted [59].

#### 2.1.3. *CPAMD8*-Associated Anterior Segment Dysgenesis

It should be noted, however, that other mutated genes have been implicated in this spectrum that account for the remaining 40% of cases other than ARS and PA [25]. Most recently, C3 and PZP-like alpha-2-macroglobulin domain-containing protein 8 (*CPAMD8*) was found to cause autosomal recessive anterior segment dysgenesis [60]. *CPAMD8* is a protein that has been documented in corneal endothelial cells and known to play a critical role in aqueous humor dynamics (specifically found in non-pigmented epithelium of the ciliary body) [61,62]. In the discovery, pathogenic variants of *CPAMD8* were a collection of missense, frameshift, and splice-site pathogenic variants. Patients across four families had unique phenotypic findings that were different than other anterior segment dysgenesis conditions. These findings included iris hypoplasia, iris transillumination defects, ectropion uveae, corectopia, iridodonesis with ectopia lentis, cataracts, normal intraocular pressure, and generally normal anterior chamber angle structure [60]. Follow-up cohort studies of children with childhood or juvenile open-angle glaucoma were found to have a low frequency of *CPAMD8* pathogenic variants causing glaucoma, especially when proband families were otherwise asymptomatic for any glaucoma [63,64]. One study recently observed human biopsy samples from a patient’s cadaveric eye with a *CPAMD8* pathogenic variant and discovered that the anterior chamber angle was subtlety malformed (e.g., variable thickness of the corneoscleral and uveoscleral trabecular meshwork) coupled with an unusual extracellular matrix deposition (e.g., patterns of basement membrane proteins not being uniform or aligned), and trabecular meshwork cells actively undergoing apoptosis [65]. This newly discovered gene and associated pathogenic variants leading to glaucoma provide even more insight into the multiple mechanisms in which glaucoma can develop, particularly the extracellular matrix structure. However, more studies are warranted to look at aqueous outflow in these types of patients or animal models with this pathogenic variant in order to develop new pharmaceutical therapies that can outperform conventional surgical bypass therapies.

### 2.2. Metabolic Disorders

The mucopolysaccharidoses are a collection of inherited lysosomal enzyme deficiencies resulting in an accumulation of glycosaminoglycans (GAGs) throughout the body. These disorders are traditionally classified into types I-IX, originally categorized based on the affected gene and enzyme, although as genetic analysis has become more detailed, so has the classification system [66,67,68,69,70,71,72,73]. The genetic causes of mucopolysaccharidoses have been well-elucidated despite these being very rare disorders. Each subtype is caused by a deficiency in a specific lysosomal enzyme resulting in decreased GAG catabolism and subsequent accumulation in nearly all body tissues (Table 2) [66,67,68,69,70,71,72,73]. Most mucopolysaccharidoses are inherited in an autosomal recessive or X-linked manner and represent loss-of-function enzymatic deficiencies. Of note, there are racial differences in prevalence and types of pathogenic variants, as described by Khan et al. [70].

The clinical features of the various mucopolysaccharidoses all result from the intra- and extracellular accumulation of glycosaminoglycans. All nine types can result in ocular abnormalities to varying degrees, most commonly corneal opacification, retinopathy, optic nerve abnormalities, and glaucoma. Table 2 summarizes the qualitative likelihood and average age of onset of these abnormalities based on a review of the current literature [74,75,76,77,78,79,80]. Based on reported studies, corneal opacification is most commonly seen in type I and type VI [74,75,76,77,78,79,80]. Continuous deposition of GAGs within the corneal stroma causes a disruption of the normally highly ordered collagen fibrils, resulting in difficult-to-treat clouding. Chronic corneal edema can lead to neovascularization and permanent scarring of the cornea, resulting in decreased vision. Poor corneal clarity also limits the ability to thoroughly examine intraocular structures such as the lens or retina. The mucopolysaccharidoses were informally categorized into the “severe” and “milder” subtypes. The “severe” subtypes (IH, II, and VII) present early in life with systemic abnormalities including facial dysmorphism and respiratory disease. The “milder” subtypes (IS, II, and IV) have less predictable and milder signs; ophthalmologists are often consulted to check for corneal clouding to aid in the diagnosis [79]. Corneal changes are treated surgically with a full thickness transplant, with some reports suggesting re-opacification in less than one year [76]. Future treatments may include local injection of adenovirus vectors within the cornea in order to reduce the accumulation of GAGs in vivo [72,73,77,79].

Previous reports are disparate regarding which mucopolysaccharidoses carry a risk of glaucoma, although there is consensus that all forms carry some glaucoma risk [71,74,75,76,77,78,79,80,81]. As such, all patients with mucopolysaccharidosis should be screened regularly for an increase in intraocular pressure or changes to the optic nerve. The mucopolysaccharidoses cause a mixed mechanism glaucoma with features of open- and closed-angle forms. There is accumulation of GAGs obstructing an otherwise open-appearing trabecular meshwork and narrowing of the anterior chamber angel from GAG accumulation within adjacent structures. Monitoring these patients is difficult, as corneal opacification limits the view into the anterior chamber and can reduce the accuracy of intraocular pressure measurements. These patients are highly resistant to medical treatment and almost invariably require surgical intervention [78]. GAG deposition within the optic nerve head commonly results in an elevated appearance, easily confused with papilledema from increased intracranial pressure. As these patients continue to live longer, it is possible that the chronic and long-term deposition within the optic nerve head may result in an optic atrophy that can cause further vision loss.

Currently, there are no routine screening protocols in infancy, although trials for type I are underway in several countries [69]. This allows for early diagnosis, frequent monitoring, and timely intervention. Additionally, past treatment for the mucopolysaccharidoses have been quite limited, resulting in a shortened lifespan and severe developmental delay. Recent implementation of bone marrow transplantation and enzyme-replacement therapy has extended the life expectancy for these patients. Because of this, ophthalmologists are just beginning to encounter long-term sequelae of these diseases. Future studies should start to elucidate ideal treatments for these patients.

### 2.3. Aniridia

Congenital aniridia is a rare disorder affecting between 1:40,00 and 1:100,000 births [82,83,84]. It is named for the partial or total hypoplasia of the iris, but aniridia is known to affect all ocular structures often resulting in corneal scarring, cataracts, glaucoma, and foveal hypoplasia. Visual acuity varies from normal to no light perception, depending on the severity and duration of symptoms.

Aniridia is categorized into sporadic (33% of cases) and autosomal dominant familial forms (66% of cases). Most forms of aniridia are caused by pathogenic variants affecting *PAX6*, a highly conserved “master regulator” of eye development, localized to 11p13 [85,86,87], with a small fraction of cases remaining unexplained or due to pathogenic variants in *ITPR1*, *FOXC1*, or *PITX2* [88]. *PAX6* contains 14 exons with upstream and downstream regulator regions [85,89]. The *PAX6* protein is a transcription factor abundantly expressed in neural and ocular tissues, thought to play a crucial role in embryogenesis. Several studies have shown no significant difference in types of pathogenic variants or the severity of disease between these groups [86]. To date, almost 500 unique *PAX6* pathogenic variants have been identified (Human *PAX6* Pathogenic variant Database, http://lsdb.hgu.mrc.ac.uk/home.php?select_db=PAX6, accessed on 29 July 2021). The majority of the submitted variants involve nonsense or frameshift pathogenic variants resulting in premature termination codons and haploinsufficiency [85,86] and these represent 75–80% of aniridia cases. Numerous studies have described *PAX6* pathogenic variants as resulting in a complete penetrance of aniridic ocular changes; however, single pathogenic variants result in a wide variety and severity of phenotypes [90,91]. Some reports have suggested that the level of disease severity is possibly correlated with the level of *PAX6* expression with loss of function pathogenic variants resulting in the most severe cases [86]. Still, this does not explain the varying severity and onset of pathology amongst family members with identical pathogenic variants [90]. Compound heterozygous pathogenic variants in *PAX6* are thought to be lethal; however, studies have described such patients with mortality in late gestation or shortly after birth with severe craniofacial and ocular malformations [85]. Of note, pathogenic variants in 11p13 have also been associated with non-aniridia ocular diagnoses, including microphthalmia, microcornea, coloboma, morning glory optic nerve, and Peters anomaly (discussed earlier). These cases are thought to be caused by missense pathogenic variants, compared with truncating pathogenic variants more frequently resulting in aniridia.

For those with sporadic aniridia, 33% of patients are diagnosed with WAGR syndrome, a contiguous gene deletion syndrome involving *PAX6* and *WT1*. These two genes are separated by 700 kb and WAGR is often caused by large deletions or rearrangements [86,92]. Of note, patients with sporadic aniridia require monitoring for the development of a Wilms tumor as part of WAGR syndrome [85,86]. Moreover, while less common, patients with pathogenic variants in *PAX6* regulatory regions have been described as well. There are cis- and trans-regulatory regions that span hundreds of kilobases up and downstream [85,86]. Furthermore, various other genes have been described as influencing iris anatomy to various degrees including *FOXC1*, *PITX2*, *CYP1B1*, *FOXD3*, and *TRIM44* [92]. Additionally, a rare cause of aniridia is the Gillespie syndrome, which features partial aniridia, cerebellar ataxia, and intellectual disability, and is caused by recessive pathogenic variants in the *ITPR1* gene [93,94].

Clinically, symptoms of aniridia can be categorized into two broad categories: congenital abnormalities and progressive changes, both of which affect vision. Congenital abnormalities including optic nerve hypoplasia and foveal hypoplasia are found in up to 20% and 90% of individuals, respectively [86]. These both affect vision at birth and are seen in association with congenital nystagmus, which is indicative of early and often permanent visual impairment. Over time, numerous additional progressive changes are seen as well. A congenital limbal stem cell deficiency results in a progressive keratopathy, resulting in dense corneal opacifications (seen in up to 80% of patients [86,95,96]). Cataracts develop in 40–80% of patients; these lens changes are often seen during childhood but do not become visually significant until young adulthood [89,97]. Possibly the most visually devastating effect on patients is glaucomatous damage to the optic nerve. Aniridic glaucoma is a combined open- and closed-angle glaucoma which is often resistant to treatment.

Systemically, patients with sporadic aniridia are at risk of WAGR syndrome, resulting in a Wilms tumor, aniridia, genitourinary abnormalities, and developmental delay. Approximately one third of patients with sporadic aniridia ultimately develop WAGR syndrome due to a contiguous gene deletion syndrome involving both *PAX6* and *WT1*. These patients have a 50% chance of developing a Wilms tumor and require an abdominal ultrasound to screen for tumor development. Gillespie syndrome, discussed above, makes up less than 2% of all cases of aniridia and is often associated with atypical ocular findings such as ptosis or corectopia [85,86,98].

Patients with aniridia need eye care throughout their entire lives. Early intervention is crucial to minimize the effects of amblyopia. Children may require spectacle correction or part-time occlusion. As patients age, multiple surgeries are all but inevitable, including cataract extraction, corneal transplants, and glaucoma surgeries. In one of the largest cohort studies of aniridia to date, trabeculectomy was found to have a 24% success rate, goniotomy/trabeculotomy were found to have a 33% success rate, and glaucoma drainage devices were found to have a success rate between 63 and 88% (manuscript in preparation).

Because of these long-term disease sequelae, the likelihood of visual impairment, and the risk of systemic disease associated with WAGR or Gillespie syndrome, genetic testing is commonly obtained for these patients. Testing usually involves sequencing of *PAX6* and *WT1*, and chromosomal microarray. Sporadic cases of aniridia are typically screened with a renal ultrasound if prior genetic testing is unavailable.

### 2.4. Collagen Vascular Disorders

#### 2.4.1. Stickler Syndrome

Stickler syndrome was originally described by Gunnar B. Stickler and his colleagues in 1965 in a select group of patients [99]. Over the subsequent 55 years, genetic testing has uncovered at least five variants of Stickler syndrome, each with unique pathogenic variants and inheritance patters. Two variants (type 1 and type 2) are autosomal dominant, resulting on nonsense or frame-shift pathogenic variants of *COL2A1* [100,101] or a missense pathogenic variant in *COL11A1* [102,103,104,105], respectively, and represent 75–90% of all cases [106]. Another two variants (type 4 and type 5) are autosomal recessive and result in nonsense pathogenic variants of *COL9A1* [107,108] and loss-of-function pathogenic variants in *COL9A2* [109], respectively, representing 10-20% of all cases [106].

Clinically, patients with Stickler syndrome have several ocular findings including myopia [110,111], cataracts (wedge shaped, 50% of patients) [112,113], and retinal detachments (with a two-fold rate of giant retinal tears in at least one eye as compared with other retinal breaks) [114]. These patients also develop glaucoma by several mechanisms. Anatomically, patients can have long iris ciliary processes that cover the trabecular meshwork, thus blocking the aqueous humor outflow from the anterior chamber and leading to intraocular pressure rise [115]. In other families, patients do not have observable anatomical abnormalities but have elevated intraocular pressure with demonstrated glaucomatous progression [116]. Of note, the above clinical characteristics of Stickler syndrome have some overlap with other common systemic syndromes including Marshall syndrome, Wagner syndrome, Weissenbacher syndrome, and Pierre Robin syndrome [101,110,117].

Genetic testing for Stickler syndrome is limited due to the complexity and number of pathogenic variants [101,110]. For most physicians evaluating potential patients, suspicion and evaluation for Stickler syndrome should be increased for the following conditions: neonates present with Pierre Robin syndrome or a midline cleft, infants with myopia/deafness/spondyloepiphyseal dysplasia, families with a history of rhegmatogenous retinal detachments, and sporadic cases of retinal detachments [101]. For treatment, prophylactic laser treatment of the retina has demonstrated a success rate of greater than 75% for preventing retinal detachments [118,119]. There is likely an underreporting of glaucoma due to the paucity of patient reports and studies in the literature [120,121]. However, for the cases that exist, most providers address lowering the pressure with filtration surgery such as goniotomy or a tube shunt. Glaucoma outcomes are typically poor in these patients, and early recognition and prompt management will be critical in the future [122].

#### 2.4.2. Osteogenesis Imperfecta

Osteogenesis imperfecta (OI) is a predominantly autosomal dominant (90% of cases) disease affecting type 1 collagen levels or structure [123]. Until very recently, four types of OI were identified with pathogenic variants in *COL1A1* or *COL1A2* inherited in an autosomal dominant manner [123], with a fifth type reported in patients with a mutated *IFITM5* gene [124]. The OI type 1 pathogenic variant results in roughly 50% less total collagen I production by cells, OI type 2 is lethal and results in death in utero, and OI types 3 and 4 have structural abnormalities of the collagen molecules [123]. Systemic findings in most patients with OI include bone fragility, low bone mineral density, skeletal deformities, dentinogenesis imperfecta, hyperlaxity of ligaments, cardiovascular disease, and hearing loss [125,126,127,128]. However, type 5 OI has bone fragility, interosseous membrane mineralization, and hyperplastic callus formation [124].

Many ocular symptoms are present in OI (Table 1). For example, corneal thickness is decreased with blue sclera more in OI type 1 [129,130,131,132,133,134], as well as lower ocular rigidity and shorter axial length [135,136]. Retinal detachments are also more frequent in patients with OI type 3 [137,138,139,140,141]. With respect to glaucoma, many patients have undermeasured intraocular pressure due to the altered corneal and scleral biomechanics, which results in patients developing glaucoma with apparently normal pressure measurements [132]. These patients are typically older (adult) and have primary open-angle glaucoma. However, in several cases for children or infants, infantile-onset glaucoma can develop and go unnoticed due to challenges in intraocular pressure measurement [123,142]. Characteristics found in younger patients with infantile-onset glaucoma (or sometimes referred to as primary congenital glaucoma) included buphthalmos, phthisis, corneal opacity, corneal edema, Haab’s striae, thin irides and elevated intraocular pressures [142]. Elevated pressure is thought to be due to gonio-trabeculodysgenesis, thus forming suboptimal trabecular meshwork and impairing aqueous humor outflow from the anterior chamber [143]. 

At present, there is no formal diagnostic criteria or genetic testing used to diagnose OI, let alone the ophthalmic findings to make an immediate OI diagnosis. Much depends on clinical suspicion and general diagnostic testing (e.g., skeletal survey). Treatment for these patients begins with conservative therapy such as aqueous suppressant eye drops, but surgical intervention can include goniotomy, trabeculotomy, trabeculectomy, or tube shunts.

#### 2.4.3. *COL4A1*

In 2015, researchers summarized 21 pathogenic variants in *COL4A1* in several families that resulted in abnormally elevated pressures leading to glaucoma [144]. Many pathogenic variants had variable expressivity and intrafamilial variability, but these were inherited in an autosomal dominant fashion. Patients with *COL4A1* pathogenic variants present with a variety of cortical abnormalities including cerebrovascular, cardiac, renal, and muscular abnormalities. With respect to the eyes, these patients harbored features similar to Axenfeld–Rieger syndrome (reviewed earlier), specifically abnormal anterior chamber angles, iris hypoplasia, eccentric pupil, iridocorneal tissue adhesions and aqueous drainage structure abnormalities coupled with elevated intraocular pressure. Because this pathogenic variant discovery is recent and novel, there are few patients to study in order to determine the mechanism driving elevated pressure and likely development of glaucoma. One mouse model study showed that mutant *COL4A1* had a dose-dependent impact on anterior segment dysgenesis coupled with abnormally elevated intraocular pressures, thus suggesting basement membrane abnormalities can be driving the phenotype [145]. This is particularly relevant as the trabecular meshwork contains this type of basement membrane, thus hypothetical treatment to prevent development or progression of glaucoma can be addressed with aqueous suppressant medications or trabecular meshwork-modifying surgeries.

#### 2.4.4. *TEK/ANGPT1*

Similar to *COL4A1*, loss-of-function pathogenic variants in *TEK* were also recently uncovered in 2016 and have a significant role in Schlemm’s canal and trabecular meshwork structure and function [146]. A cohort of 189 families were studied and 10 heterozygous pathogenic variants leading to haploinsufficiency were documented, along with observations of non-penetrance and variable expressivity [146]. Patients with these pathogenic variants were found to have primary congenital glaucoma due to malformation of the aforementioned structures, especially when at least 50% of *TEK* signaling was reduced. Mouse models have also confirmed these findings [146,147,148]. Additionally, variants in *ANGPT1*, a ligand for *TEK*, also resulted in a similar abnormal Schlemm’s canal and trabecular meshwork abnormalities and led to the development of congenital glaucoma [147]. Heterozygous pathogenic variants in *ANGPT1* similarly have low penetrance, with two thirds of carriers within families being asymptomatic. This creates both diagnostic and therapeutic challenges in identifying patients that are at high risk of glaucoma and choosing the appropriate treatment based on precise outflow pathway anatomy. Because the drainage pathway is affected, it can be speculated that similar aqueous suppressant medications or trabecular meshwork-modifying surgeries may best address this form of glaucoma. However, if downstream collector channels are affected, then tube shunts or trabeculectomies that bypass the traditional outflow pathways entirely would be more effective.

### 2.5. Immunogenetic Disorders Associated with Glaucoma

Mendelian immunogenetics disorders are a class of monogenic disorders that lead to disease pathology through the disruption of immune pathways and activation of autoimmunity or autoinflammation. Glaucomatous optic neuropathy is a common feature of several of these conditions and two prototypical conditions are Aicardi–Goutieres syndrome (AGS) and Singleton–Merten syndrome (SGMRT).

AGS was first described by Jean Aicardi and Francoise Goutières in 1984 with a case series of eight children from five families with severe early-onset encephalopathy [149]. AGS can be inherited as an autosomal dominant or recessive condition and has significant allelic heterogeneity. AGS is caused by pathogenic variants in genes involved in RNA processing (*TREX1*, *RNASEH2A*, *RNASEH2B*, *RNASEH2C*, *SAMHD1*, and *ADAR*) and innate immunity (*IFIH1*) (Table 1) [150,151,152]. In the most severe form, this condition features microcephaly, leukodystrophy, cerebral atrophy, intracranial calcifications, along with hepatosplenomegaly and thrombocytopenia. It is associated with elevated levels of central nervous system type I interferon signaling and often progresses with severe neurologic symptoms and death in early childhood. However, milder forms may show later onset with features of a lupus-like syndrome including painful skin lesions and congenital glaucoma. In a large study on AGS, glaucoma was reported in 6.3% (23 patients), including 20.8% (10/48 patients) with *SAMHD1* pathogenic variants and no patients with pathogenic variants in *ADAR* or *IFIH1* [153].

Singleton–Merten syndrome (SGMRT) is an autosomal dominant condition first described by Edward B. Singleton and David Merten in 1973 [154]. SGMRT is caused by gain-of-function variants in one of two RIG-I-like receptor proteins (*DDX58* or *IFIH1*) [155,156,157]. These receptors normally recognize exogenous double-stranded RNA and activate innate immune pathways and type I interferon signaling as part of the antiviral response. Systemic features of SGMRT include a psoriasiform skin rash, vascular calcifications, skeletal dysplasia, and dental anomalies. There is variable expressivity, but the prominent ocular feature is juvenile open-angle glaucoma. Glaucoma is the most penetrant feature of SGMRT caused by *DDX58* variants, present in 17/18 (94%) of reported cases [155,158,159]. However, there is both intra-familial and interfamilial variability in the age of onset and severity, with median age of diagnosis at 5 years of age (range 2–18 years of age). *IFIH1*-related SGMRT also features glaucoma in a smaller fraction of cases (~40%) and up to 13.5% of pathogenic variant carriers can be asymptomatic [153]. Additionally, patients with SGMRT may have an elevated rate of corneal transplant failure and have an ocular surface disease [158].

While patients with congenital and juvenile glaucoma secondary to immunogenetic disorders have typically been managed in a similar way as their primary counterparts, the systemic features of AGS have been responsive to immunomodulatory therapy. Specifically, Janus kinase (JAK) inhibitors such as baracitinib hold significant promise in reducing the neurological features of Aicardi–Goutieres, though randomized-controlled trials are needed to substantiate these effects [160]. While these therapies have not been systematically attempted for SGMRT, there is evidence from animal models that they may be also effective for this condition [161]. It is unclear whether these immunomodulatory therapies can be useful for the management of glaucoma associated with these conditions and that remains a topic of investigation.

### 2.6. Nanophthalmos

Nanophthalmos is a heritable condition characterized by a small, but structurally normal eye, with resultant high hyperopia. Defining features of nanophthalmos includes a short axial length, variably defined in the literature as less than 20-21 mm, with a proportional decrease in anterior segment dimensions, i.e., corneal diameter and anterior chamber depth [162]. Hyperopia is a strongly heritable genetically with twin studies suggesting that 70–90% of variance in this disorder is attributable to genetic causes [163,164]. Five major genes have been implicated in isolated nanophthalmos, including *MFRP, PRSS56, BEST1, TMEM98, CRB1,* and *MYRF* [162,165,166,167,168,169]. For two of these genes (*TMEM98*, *MYRF*), the trait is inherited in an autosomal dominant manner, while the remaining genes cause recessive disease (*MFRP*, *PRSS56*, *BEST1*, *CRB1*). Additionally, several genes have been associated with nanophthalmos as part of a multisystem syndrome, including *MYRF* and *FAM111A* [168,170]. Variants in *MYRF*, which underlies the NNO1 locus [168,171], have been identified an emerging syndrome featuring a congenital diaphragmatic hernia, cardiac and pulmonary vascular anomalies, urogenital anomalies, and nanophthalmos [168,172,173]. Kenny–Caffey syndrome is a rare autosomal dominant syndrome caused by pathogenic variants in *FAM111A* and featuring skeletal dysplasia, short-stature, and microorchidism in males [170,174,175]. Cohort studies of nanophthalmos have had a widely variable diagnostic yield of genetic testing between 19 and 90% [176,177,178]. This is likely related to founder effects, patient population selection, and differences among ethnic groups. Defining the genetic etiology is important for patient counseling and management as different genetic causes are associated with varying ocular and systemic features.

Though the defining feature of nanophthalmos is short axial length, nanophthalmos is frequently associated with ocular complications, presenting as high hyperopia with amblyopia and partially accommodative esotropia in early childhood (Table 1) [162]. Given the anterior segment structure, patients are predisposed to angle closure and the resulting angle closure glaucoma, and often need cataract surgery early in life. Vision loss may also result from other associated retinal findings, including foveal hypoplasia, optic disc drusen, retinoschisis and foveoschisis, retinitis pigmentosa, chorioretinal folds, or central retinal vein occlusions, or complications from ocular surgery [162,179]. *MFRP, CRB1,* and *BEST1* genes have also been variably associated with other ocular features including retinal degeneration, optic disc drusen, and macular dystrophy [180,181,182]. Many genes associated with Leber congenital amaurosis in addition to *CRB1* are also associated with high hyperopia and short axial length [183,184,185].

Glaucoma and other ocular complications secondary to nanophthalmos may be difficult to manage and genetic diagnosis can be helpful for early recognition. For example, in patients with cardiac-urogenital syndrome and *MYRF* loss-of-function variants, an early screen for refractive error and amblyopia is critical to prevent irreversible vision loss. Angle closure in nanophthalmos patients is primarily managed by lens extraction, but complications can occur in up to 40–60% of cases [179,186,187]. Intraoperative and post-operative risks include increased rates of corneal endothelial damage, capsular rupture and vitreous loss, intraoperative aqueous misdirection, uveal effusion syndrome, and cystoid macular edema. Prophylactic scleral windows may reduce the rate of complication, owing to a thickened sclera and choroid that may predispose these conditions. While genetic therapies for nanophthalmos have not yet reached the clinical realm, there is some promise for gene replacement strategies for *MFRP* based on animal models [188].

## 3. Conclusions

Childhood glaucoma has many associations with systemic syndromes that have grounding in key regulatory genes. While some types of childhood glaucoma, such as ARS or aniridia, have hundreds of known pathogenic variants in a single gene, others, such as nanophthalmos and collagen-vascular disorders, have significant genetic heterogeneity. Diagnostic yield from genetic testing thus varies widely for each of the conditions. However, many of the specific constellations of ocular and systemic signs and symptoms may point to a specific genetic diagnosis in the hands of an astute clinician. Furthermore, recognition of these syndromes is critical for early diagnosis and treatment of glaucoma associated with these disorders. It is fortunate that many of these syndromes yield consistent clinical findings, as it would otherwise be difficult to detect elevated intraocular pressure or glaucoma in children given difficulty in communication and clinical testing. These consistent features can be attributed to the fact that the regulatory genes that are commonly affected in childhood glaucoma ultimately play a role in other developmental processes and cause perturbations in other organ systems. Even more, while rarer forms of childhood glaucoma have more severe and obvious deficits, their underpinnings may shed light on the possible mechanisms driving other forms of glaucoma (e.g., adult-onset glaucoma) with subtle side effects. Genetic testing plays a critical role in confirming clinical diagnostic suspicions and will become more important as gene/disease specific treatments are developed for childhood glaucoma.

Fortunately, the ability to create targeted biopharmaceutics to address these pathogenic variants are coming within reach due to the advent of high-throughput technologies such as single-cell RNA sequencing, whole genome sequencing with small sample inputs, CRISPR/Cas9 gene editing technology, compound library drug screening, and large scale hybridoma antibody synthesis. The dramatic drop in the cost of these technologies and high-fidelity of their performance can herald a new era of expedited diagnoses of childhood glaucoma coupled with possible treatments that mitigate or slow the rate of vision loss. Furthermore, specific rare syndromes can shed light on common biological mechanisms of glaucoma and novel pathways to explore for therapeutics, including bypassing specific structural defects and immunomodulatory therapy. However, the limited study population of patients and families with childhood glaucoma presents a challenge for therapeutic trials. For many of the above subtypes of childhood glaucoma, the description was completely dependent on the findings multi-generational families with highly penetrant variants of syndrome-associated glaucoma. Unfortunately, without more study subjects that have clear inheritance and pathogenic variant patterns and better descriptions of natural history and outcomes, it will remain difficult to expeditiously advance future diagnostics and therapeutics. Our review highlights the value that studying these rare disorders can bring towards patient diagnosis, treatment, and providing insights into more common forms of glaucoma.

## Figures and Tables

**Table 1:** Features of glaucoma syndromes.

Syndrome	Known Genes (Inheritance Pattern)	Ocular Features	Systemic Features
**Anterior segment dysgenesis (including Axenfeld–Rieger syndrome)**	*PITX2* (AD) *FOXC1* (AD) *CPAMD8* (AR)	Posterior embyotoxon, corectopia, iris hypoplasia, polycoria, dysplastic angle structures, glaucoma *CPAMD8* with also iridodonesis, ectopia lentis, ectropion uvea	Axenfeld–Rieger syndrome: dental hypoplasia, flat mid-face, umbilical abnormalities, pituitary abnormalities, cardiac defects, sensorineural hearing loss, myotonic dystrophy
**Peters Anomaly**	PITX2 (AD) FOXC1 (AD) *PAX6* (AD) *CYP1B1* (AD/AR) *B3GALTL* (AR)	Central corneal opacities, iris synechiae, absence of corneal endothelium, absence of descemet membrane, glaucoma	Peters Plus syndrome: short stature, abnormal ears, brachyomorphism
**Aniridia**	*PAX6* (AD/AR) *ITPR1* (AD/AR) *FOXC1* (AD) *PITX2* (AD)	Iris hypoplasia (total or partial), limbal stem cell deficiency, keratopathy, cataracts, foveal hypoplasia, optic nerve hypoplasia, nystagmus, glaucoma	WAGR syndrome: Wilms tumor, genitourinary abnormalities, mental retardation Gillespie syndrome: Cerebellar ataxia, intellectual disability
**Stickler Syndrome**	*COL2A1* (AD) *COL11A1* (AD) *COL9A1* (AR) *COL9A2* (AR)	Myopia, cataracts, retinal detachments, elongated ciliary processes, glaucoma	Midface hypoplasia, cleft palate, glossoptosis, sensorineural hearing loss, short stature, arthropathy
**Osteogenesis Imperfecta**	*COL1A1* (AD) *COL1A2* (AD) *IFITM5* (AD)	Corneal thinning, scleral thinning (blue sclera), low ocular rigidity short axial length, retinal detachment, glaucoma	Bone fragility, low bone mineral density skeletal deformities, dentinogenesis imperfecta, hyperlaxity of ligaments, cardiovascular disease, hearing loss
***COL4A1*-associated connective tissue disorder**	*COL4A1* (AD)	Anterior segment dysgenesis similar to Axenfeld–Rieger syndrome	Cerebrovascular abnormalities, leukoencephalopathy, cardiac abnormalities, renal abnormalities, muscular abnormalities
***TEK/ANGPT1*—glaucoma**	*TEK* (AD) *ANGPT1* (AD)	Primary congenital glaucoma	None
**Aicardi–Goutieres Syndrome**	*TREX1 (AR)* *RNASEH2A (AR)* *RNASEH2B (AR)* *RNASEH2C (AR)* *SAMHD1 (AR)* *ADAR (AR)* *IFIH1 (AD)*	Congenital glaucoma, optic atrophy, cortical blindness	Encephalopathy, microcephaly leukodystrophy, cerebral atrophy, intracranial calcifications, hepatosplenomegaly, thrombocytopenia, lupus-like syndrome
**Singleton–Merten Syndrome**	*DDX58 (AD)* *IFIH1 (AD)*	Congenital or juvenile open-angle glaucoma Ocular surface disease	Psoriasiform rash, vascular calcifications skeletal dysplasia, tendon rupture, arthritis, dental anomalies
**Nanophthalmos**	*MFRP (AR)* *PRSS56 (AR)* *BEST1 (AR)* *TMEM98 (AD)* *CRB1 (AR)* *MYRF (AD)* *FAM111A (AR)*	Axial hyperopia, esotropia, foveal hypoplasia, optic disc drusen, retinoschisis/foveoschisis, retinitis pigmentosa, chorioretinal folds, central retinal vein occlusions, angle closure glaucoma	Cardiac-urogenital syndrome (CUGS): Diaphragmatic hernia, cardiopulmonary vascular anomalies (i.e., Scimitar syndrome), pulmonary hypoplasia, urogenital anomalies Kenny-Caffey syndrome: skeletal dysplasia, short stature, hypocalcemia, microorchidism

AD: autosomal dominant; AR: autosomal recessive.

**Table 2:** Clinical and molecular features mucopolysaccharidosis subtypes.

Type	Subtype	Eponym	Defective Enzyme	Accumulated GAG	Gene Locus	Inheritance	Corneal Clouding	Glaucoma	Optic Neuropathy
I	IH	Hurler	α-L-Iduronidase	HS, DS	4p16.3	AR	+ → +++ 6 months–1.1 years	+/++ 1 year	+/++ 17 years
	I H/S	Hurler-Scheie	α-L-Iduronidase	HS, DS	4p16.3	AR	+/++ 4.4 years	++ 1 year	++ 17 years
	IS	Scheie	α-L-Iduronidase	HS, DS	4p16.3	AR	+ → +++ 24 months–10.5 years	+/++ 1 year	+/++ 17 years
II		Hunter	Iduronate-2-sulfatase	HS, DS	Xq28	XL recessive	Clear/+	+ 7.5 years	None → ++ 33 years
III	A	Sanfilippo A	Heparan-N-sulfatase	HS	17q25.3	AR	+	+	+
	B	Sanfilippo B	α-N-acetylglucosaminidase	HS	17q21	AR	+	+	+
	C	Sanfilippo C	α-glucosaminidase-acetyltranferase	HS	8p11.1	AR	+	+	+
	D	Sanfilippo D	N-acetylglucosamine-6-sulfatase	HS	12q14	AR	+ 8 years	+	+
IV	A	Morquino A	N-acetylgalactosamine-6-sulfatase	KS	16q24	AR	+ 11 years	+ 7.8 years	None → +
	B	Morquino B	β-galactosidase	KS	16q24	AR	+ 11 years	+ 7.8 years	None → +
VI		Maroteaux-Lamy	N-acetylgalactosamine-4-sulfatase	DS	5q12	AR	+++ 7 years	++ 3 years	None → ++ 26 years
VII		Sly	β-D-glucuronidase	HS, DS, KS	7q22	AR	+/++ 15 years	++	None → ++
IX		Natowicz	Hyaluronidase	CS	3p21.2–3	AR	unknown	unknown	unknown

GAG: glycosaminoglycan, DS: dermatan sulfate, KS: keratan sulphate, CS: chondroitin sulphate, AR: autosomal recessive, XL: X-linked.

## Data Availability

All relevant data are included in the manuscript text and figures.
